# Altered cognitive control network is related to psychometric and biochemical profiles in covert hepatic encephalopathy

**DOI:** 10.1038/s41598-019-42957-6

**Published:** 2019-04-29

**Authors:** Chia-Fen Tsai, Pei-Chi Tu, Yen-Po Wang, Chi-Jen Chu, Yi-Hsiang Huang, Han-Chieh Lin, Ming-Chih Hou, Fa-Yauh Lee, Pei-Yi Liu, Ching-Liang Lu

**Affiliations:** 1 Institute of Brain Science, National Yang-Ming University School of Medicine, Taipei, Taiwan; 2 Faculty of Medicine, National Yang-Ming University School of Medicine, Taipei, Taiwan; 3 Division of Gastroenterology, Taipei Veterans General Hospital, Taipei, Taiwan; 4 Endoscopy Center for Diagnosis and Treatment, Taipei Veterans General Hospital, Taipei, Taiwan; 5 Department of Medicine, Taipei Veterans General Hospital, Taipei, Taiwan; 6 Department of Psychiatry, Taipei Veterans General Hospital, Taipei, Taiwan; 7 Department of Medical Research, Taipei Veterans General Hospital, Taipei, Taiwan

**Keywords:** Encephalopathy, Brain, Liver cirrhosis

## Abstract

The cognitive control network (CCN) is a network responsible for multiple executive functions, which are impaired in covert hepatic encephalopathy (CHE). We aimed to use functional connectivity (FC) magnetic resonance imaging to test the hypothesis that CHE manifested with disconnection within the CCN, which is associated with impaired neuropsychiatric and biochemical profiles. CHE was detected with abnormally low psychometric hepatic encephalopathy scores (PHES) (total cut-off score <−4). Two seeds in the dorsal anterior cingulate cortex (dACC) and the dorsolateral prefrontal cortex (DLPFC) were used to calculate the FC map within the CCN. Pearson correlation analysis was performed between the CCN and psychometric, biochemical profiles including ammonia, Interleukin (IL)-6, and tumor necrosis factor (TNF)-α. Eighteen CHE, 36 non-HE (NHE) cirrhotic patients and 36 controls were studied. Significant differences in FC were noted among groups, which revealed CHE patients had a lower FC in the bilateral lateral occipital cortex (seed in the bilateral dACC) and in the right lateral occipital and precuneus cortices (seed in the left DLPFC) (P < 0.05, corrected) compared with NHE. Progressively decreased FC in the left precentral gyrus within the CCN was noted from control, NHE to CHE. PHES positively and biochemistry negatively correlated with FC in the CCN. In conclusion, CHE patients showed aberrant FC within the CCN which is correlated with both cognitive dysfunction and biochemical profiles. Ammonia and pro-inflammatory cytokines may contribute to the occurrence of aberrant connectivity. Impaired FC within the CCN may serve as a complementary biomarker for CHE.

## Introduction

Hepatic Encephalopathy (HE), ranging from subtle alterations to stupor and coma, is a common and serious neuropsychiatric syndrome that occurs in cirrhotic patients. Covert hepatic encephalopathy (CHE) is considered as the pre-clinical stage of overt HE and has received increased attention due to its negative impacts, such as impaired driving skills, decreased quality of life, increased falls and mortality^1^. CHE is associated with a series of brain-related functional changes, and the source of this bias may be complex. Reduced resting-state functional connectivity with respect to attention, such as default mode network (DMN), dorsal attention network (DAN) and anterior cingulate cortex (ACC), has been associated with cognitive dysfunction in CHE compared to controls^2–4^. Studies using magnetoencephalography (MEG) have also revealed that tremor-like symptoms in HE are associated with slowing of oscillatory cortico-muscular^5,6^ and thalamo-cortical coupling^7^. This oscillatory neuronal slowing in the motor system is paralleled by neuronal slowing in the visual system reflected as reduced critical flicker frequency test^5^ and suggested that slowing of oscillatory processes represents a common pathophysiological mechanism underlying diverse clinical symptoms in HE^8–10^.

Although attentional dysfunction has been proposed as the fundamental cognitive impairment in CHE^11^, the neurocognitive manifestations of CHE are actually much more complicated. In addition to attention, multi-facet neuropsychological abnormalities, including visual tracking, working memory, and executive function, have been recognized in CHE patients^12^.

The cognitive control network (CCN) modulates executive functions, including selective attention, working memory, stimulus-response mapping and performance monitoring^13^. The CCN has also been supposed to support switching between different mental representations and is a strong candidate to mediate attention-memory interactions^14,15^. The aforementioned cognitive dysfunctions are commonly observed in CHE patients^12^. In addition, the CCN is also positively correlated with DMN and DAN at rest, and connectivity is impaired in HE patients^15^. Furthermore, HE patients have functional or metabolic abnormalities in the CCN-related brain regions. For example, a positron emission tomographic study revealed impaired blood flow and oxygen metabolism in the frontal cortices and ACC of HE patients^16^. Decreased activation in the ACC and bilateral prefrontal cortex (PFC) was observed in HE patients while performing incongruous color-naming tasks compared with controls^17^. Furthermore, the increased severity of HE was associated with significantly reduced nodal efficiency in the frontal cortices, including the ACC and dorsal lateral PFC (DLPFC)^18^. Nevertheless, the role of the CCN in the pathogenesis of clinical manifestations of CHE remains unclear.

Ammonia has been traditionally proposed as an important biomarker for patients with overt HE^19^. Although controversial, elevated ammonia has been demonstrated to impair brain connectivity in HE patients^20,21^. Evidence has also shown that pro-inflammatory cytokines mediate the hepatic inflammation, apoptosis and necrosis of liver cells and induce cholestasis and fibrosis in patients with chronic liver diseases^19^. These cytokines can act synergistically with ammonia to affect brain function in humans and animals with cirrhosis^19^. Nevertheless, the interaction between serum markers (ammonia and pro-inflammatory cytokines) and the resting-state brain network remains unknown.

In the present study, we examine the hypothesis that the negativity bias among cirrhotic patients with and without CHE may reflect a deficit in the control of cognition, potentially associated with deficits in brain regions supporting the CCN, such as DLPFC and dorsal ACC (dACC)^22,23^. Secondarily, we explored the correlation between changes in FC within the CCN and neurocognitive performance or biochemical profiles, including ammonia and pro-inflammatory cytokines.

## Methods

### Subject and cirrhotic patient enrollment

The study protocol was approved through the institution’s review board of Taipei Veteran’s General hospital. All experiments were performed in accordance with relevant guidelines and regulations. The present study was conducted from January 2013 to Aug 2016 at an outpatient clinic in a tertiary 2,700-bed referral center in Northern Taiwan. Two samples of subjects were invited to participate in the present study. A sample of healthy adult Taiwanese (>=20 years old) was enrolled via advertisement and the other sample comprised consecutive patients with liver cirrhosis from the clinic. All cirrhotic patients received a standard clinical examination, including a general physical examination and laboratory investigations. Cirrhosis was diagnosed according to liver biopsy and/or clinical data, endoscopic findings (presence of varices), fibroscan, and ultrasonography. Liver disease severity was determined based on the Child-Turcotte-Pugh (CTP) score. The following exclusion criteria were applied: alcoholic cirrhosis; a history of recent (<3 months) alcohol intake; the presence of overt HE^1^ (grade 2 or with evidence of asterixis and disorientation); taking lactulose, antibiotics, antidepressants, or antipsychotics drugs in the preceding 8 weeks; the presence of substantial comorbidities, including heart disease, respiratory system disease, and renal failure; the presence of neurological or psychiatric diseases; and the presence of hepatocellular carcinoma or another malignancy. Advertisement posters were used to invite healthy subjects to participate in the present study as controls. The following exclusion criteria were applied for healthy controls: abnormal results for renal function; presence of chronic renal, neurological or major psychiatric diseases, or other diseases that can affect cognitive function; a history of a neurologic or psychiatric disorder; alcohol abuse (>60 g/day for men and >30 g/day for women); and a history of psychotropic drug consumption.

On the day of brain imaging, venous sampling was performed, and the sample was sent for routine blood biochemistry tests, hematological parameters, and serum cytokine levels. Written informed consent was obtained from all participants.

### Blood samples for cytokines

To obtain the blood-serum samples, 10 mL of blood was collected using BD Vacutainer tubes (Becton, Dickinson Company, NJ, USA) and centrifuged at 1000 g for 15 min. The supernatants were collected and immediately stored at −80 °C until further use. Serum IL-6 and TNF-alpha levels were determined using commercially available ELISA test kits (Bender MedSystems, Vienna, Austria)^24^.

### Neuropsychological assessment

#### Psychometric Hepatic Encephalopathy Score (PHES)

All enrolled subjects completed the five neuropsychological tests that make up the PHES (the digit symbol test (DST), the number connection test-A (NCT-A), the number connection test-B (NCT-B), the serial dotting test (SDT), and the line tracing test (LTT)). Trained medical assistants administered all tests were in the morning, and the tests were performed in a quiet room with appropriate lighting. We conducted a validation study using PHES to detect CHE in Taiwan. Differences for each subtest of PHES in multiples of the standard deviation (SD) were summed as Z scores in the following manner: a result ≤1 SD above the norm value was scored as +1; results −1 SD and −2 SDs below the norm value were scored as −1 and −2, respectively; and a result ≤−3 SDs was scored as −3. The sum of the Z scores for the PHES ranged from +5 to −15. CHE was defined whenever a total PHES <−4 was detected^25^.

#### Hospital Anxiety and Depression Scale (HADS)

We measured the degree of psychological distress using the Hospital Depression and Anxiety Scale, developed and validated for use in nonpsychiatric medical patients. The survey items related to mood disorders and physical illnesses were eliminated. For this scale, high scores indicated poor mental health^26^.

### Magnetic Resonance Imaging(MRI)

Image acquisition: Images were acquired using a 3.0 GE Discovery 750 whole-body high-speed imaging device. Head stabilization was achieved with cushioning, and all participants wore earplugs (29 dB rating) to attenuate noise. Automated shimming procedures were performed, and scout images were obtained. Resting-state functional images were collected using a gradient echo T2* weighted sequence (TR/TE/Flip = 3000 ms/30 ms/90°). Forty-seven contiguous horizontal slices, parallel to the inter-commissural plane (voxel size: 3 × 3 × 3 mm), were acquired interleaved. During functional runs, the subject was instructed to remain awake with his or her eyes open (one run, each run 6 min and 12 s, 124 time points). In addition, a high-resolution structural image was acquired in the sagittal plane using a high-resolution sequence (repetition time (TR), 2530 ms; echo spacing, 7.25 ms; echo time (TE), 3 ms; flip angle 7°) with isotropic 1 mm voxel; FOV 256 × 256.

### Analysis of resting-state FC

#### FC pre-processing

The motion-corrected functional scans received slice-timing correction and motion correction; the scans were also registered to the Montreal Neurological Institute atlas using FSL (FMRIB Software Library, www.fmrib.ox.ac.uk/fsl). Additional preprocessing steps, described in previous reports^27^, were used to prepare the data for FC analysis: (1) spatial smoothing using a Gaussian kernel (6 mm full width at half-maximum), (2) temporal filtering (0.009 Hz < f < 0.08 Hz), and (3) removal of spurious or nonspecific sources of variance by regression of four variables. The four regressed variables include the following: (a) the six movement parameters computed by rigid body translation and rotation in preprocessing, (b) the mean whole brain signal, (c) the mean signal within the lateral ventricles, and (d) the mean signal within a deep white matter region of interest (ROI). The first temporal derivatives of these regressors were included in the linear model to account for time-shifted versions of spurious variance. The regression of each of these signals was simultaneously computed, and the residual time course was retained for the correlation analysis.

#### FC analysis

Between-group comparisons of the CCN were performed using whole-brain analysis. The anatomical components of the CCN in each participant were identified based on the literature^28^, and one seed was placed in each hemisphere in the dorsal ACC (−4/4, 30, 22) and the DLPFC (−36/36, 28, 34) to identify FC within the CCN. Each seed was spherical in shape with a diameter of 8 mm. For each seed, individual participant analyses were performed using the FSL toolbox with seed-based regression approaches on residualized resting-state data^29,30^. The time series for each seed was entered as a predictor. Individual subject level, Z-statistic images were generated for each seed. Fisher’s r-to-z transformation was used to convert correlation maps into z maps^27^.

### Statistical analyses

The data are expressed as an absolute number and a percentage for categorical variables and as the mean ± SD for continuous variables. The chi-square test and the Fisher exact test were used for comparisons between the categorical variables. For continuous variables, the Mann–Whitney test and the Wilcoxon rank-sum test were used for unpaired data and for paired data, respectively.

#### Group analysis of the FC in the CCN

Within each group, a random effect one-sample *t*-test was performed on an individual Z-value map in a voxel-wise manner to determine brain regions showing significant FC to the seed region of the CCN. Significant thresholds were set at a corrected P < 0.001 with multiple sample correction using false discovery rate (FDR) criterion^31^ across the whole brain. An ANOVA test in a voxel-wise manner was then performed to determine differences in the CCN resting-state networks between CHE, NHE patients and healthy controls, with age, sex, and head motion as covariates. Differences in the mean Z-scores within the CCN resting-state networks between groups were also investigated. Post hoc two-sample *t*-tests were performed to examine differences between groups. The statistical threshold was also set at P < 0.05 and was FDR corrected.

To investigate the potential effect of venous blood ammonia and serum pro-inflammatory cytokines on resting-state networks and to examine the relationship between resting-state networks and the neuropsychological performance of cirrhotic patients, the regions within the each resting-state network that significantly differed between CHE patients and NHE groups were extracted as a mask comprising several ROIs. Then, the mean Z-values of each patient within these ROIs were correlated against the venous ammonia levels, serum cytokine levels, the Z-scores of PHES and its subdomain scales, using Pearson’s correlation analysis. The correlation analysis was performed using SPSS 16.0 (SPSS Inc., Chicago, IL), and the threshold was set at a significance level of P < 0.05.

## Results

### Demographics and clinical data

Ninety adults, including 18 CHE (63.3 ± 10.7 years), 36 NHE (57.9 ± 8.6 years), and 36 healthy subjects (59.9 ± 10.5 years), were enrolled. The demographics and clinical data of these 90 participants are summarized in Table 1. There was no significant difference in gender, age, and mood status (indicated as HADS scores) between groups (Table 1). The cirrhotic patients had lower platelet count (normal range: 150–350 × 10^3^/μL): while the CHE patients had significant lower platelet count than that in the NHE counterpart (Table 1). Cirrhotic patients had higher serum levels of IL-6 and TNF-alpha and poorer neuropsychological performance (measured using PHES) than healthy controls (Table 1). Between the 2 cirrhotic groups, CHE patients showed poorer cognitive performance in the PHES and higher serum level of IL-6 than NHE cirrhotics. No differences were noted in Child-Pugh score, venous ammonia level, and TNF-alpha level between the 2 cirrhotic patients (with and without CHE).

**Table 1:** Demographics and clinical data.

	NHEN = 36	CHEN = 18	P value	NCN = 36	ANOVAP value
Age, yrs (SD)	57.9 (8.6)	63.3 (10.7)		59.9 (10.5)	0.17
Sex (male %)	58.3%	55.6%		50%	0.77
PHES	−0.3 (1.4)	−6.9 (2.8)		−0.3 (1.6)	<0.001
HADS	8.3 (6.8)	10.8 (4.8)		9.8 (6.2)	0.32
Child-Pugh category A/B	31/5	14/4	0.46 (chia square)		
Etiology of cirrhosis HBV/HCV/Alcohol	20/10/6	9/6/3	0.91 (chia square)		
Albumin (g/dL)	3.9 (0.7)	3.5 (0.5)	0.18		
ALT (U/L)	60.1 (58.8)	50.6 (35.2)	0.50		
AST (U/L)	47.2 (36.6)	59.8 (39.3)	0.30		
PLT(10^3^/*μ*L)	111.2 (38.2)	84.8 (44.0)	0.01		
Sodium (mEq/L)	140.2 (2.5)	139.0 (2.4)	0.31		
Hemoglobin (g/dL)	13.2 (2.7)	11.7 (2.7)	0.12		
IL-6 (pg/mL)	1.9 (0.7)	3.3 (1.7)	0.007	1.6 (0.2)	<0.001
TNF-α (pg/mL)	35.7 (33.2)	60.7 (77.1)	0.23	15.6 (6.9)	0.05
Ammonia (μg/dL)	29.8 (12.3)	32.3 (15.5)	0.56		

Data was expressed as mean (SD); NC = normal control, NHE = non-hepatic encephalopathy cirrhotics, CHE = covert hepatic encephalopathy, PHES = Psychometric Hepatic Encephalopathy Score, HADS = Hospital Anxiety and Depression Scale; HBV = hepatitis B virus; HCV = hepatitis C virus; ALT = alanine aminotransferase, AST = aspartate aminotransferase, IL = interleukin, TNF = tumor necrosis factor.

### CCN resting-state functional network

**Figure 1:**
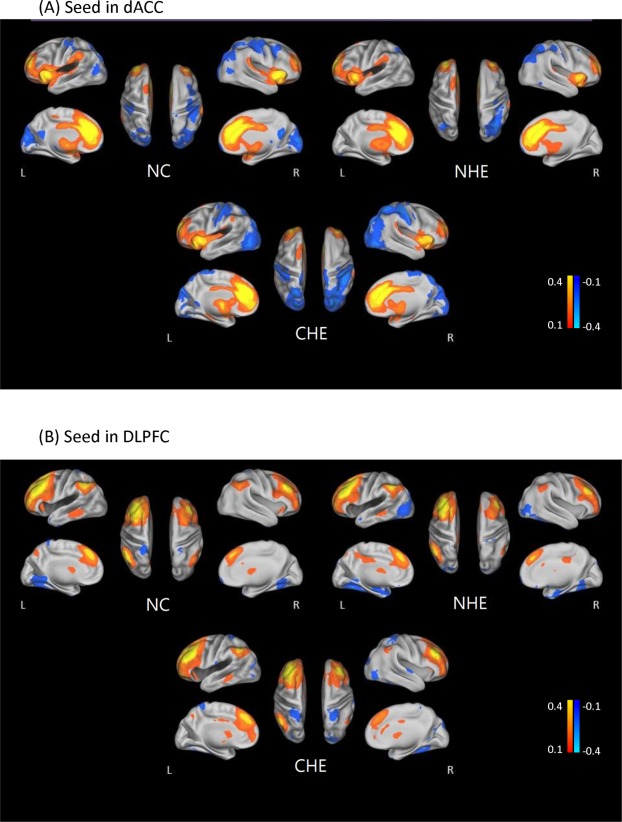
Statistical t-maps of cognitive control network resting-state connectivity in (1) NC, (2) NHE, and (3) CHE with seeds in the dorsal anterior cingulate cortex (dACC) and the dorsolateral prefrontal cortex (DLPFC). The color scale represents t-values in each resting-state network (P < 0.05, false discovery rate corrected, dACC = dorsal anterior cingulate cortex; DLPFC = dorsolateral prefrontal cortex, NC = normal controls, NHE = non-hepatic encephalopathy cirrotics, CHE = covert hepatic encephalopathy).

All healthy controls and cirrhotics showed significant FC within the CCN, including the dACC, DLPFC, supramarginal, superior parietal and inferior parietal regions, and thalamus (see Fig. 1A for seed in dACC and Fig. 1B for seed in DLPFC).

Relative to controls, the cirrhotic patients showed a decreased FC in the CCN (P < 0.001, FDR corrected). The FC patterns of the CCN were similar in the right and left hemispheres.

**Figure 2:**
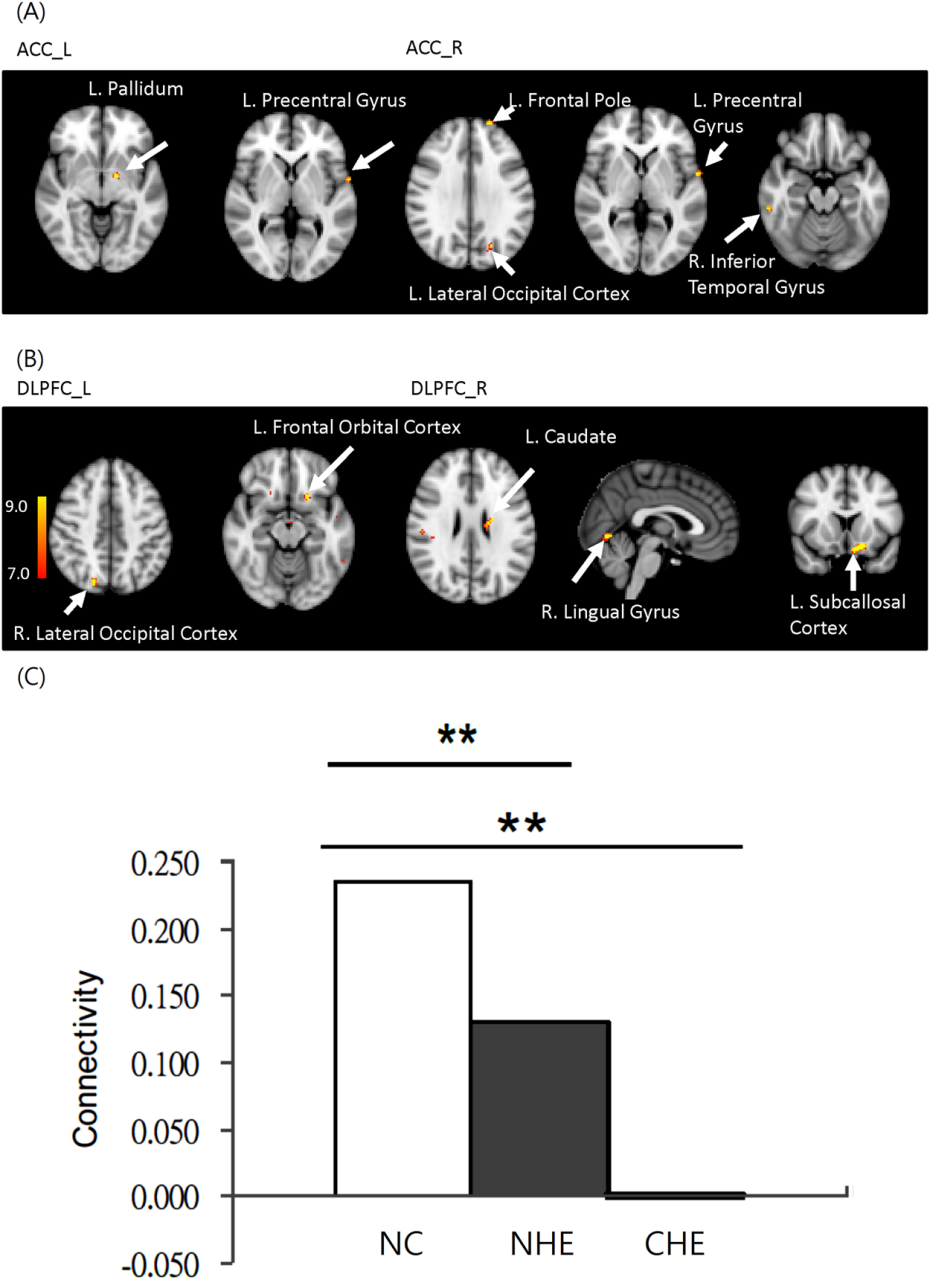
Differences in the functional connectivity within the cognitive control network among groups after an ANOVA test were noted in the (**A**) left pallidum and precentral gyrus (seed in left dACC); left frontal pole, right inferior temporal gyrus, left precentral gyrus, and left lateral occipital cortex (seed in right dACC); (**B**) left frontal orbital cortex, and right lateral occipital cortex (seed in left DLPFC); and left subcallosal cortex, right lingual gyrus and left caudate (seed in right DLPFC). (all P < 0.05, false discovery rate corrected) (**C**) There was significant severity-dependent decrease of mean cognitive control network FC in left precentral gyrus between groups (**P < 0.05) (dACC = dorsal anterior cingulate cortex; DLPFC = dorsolateral prefrontal cortex, NC = normal controls, NHE = non-hepatic encephalopathy cirrotics, CHE = covert hepatic encephalopathy)

ANOVA revealed differences in the FC among healthy controls, NHE patients, and CHE patients. There were significant differences of FC in the left pallidum and precentral gyrus when the seed was placed in the left dACC; there were significant differences in the left frontal pole, right inferior temporal gyrus, left precentral gyrus, and left lateral occipital cortex when the seed was placed in the right dACC; there were significant differences in the left frontal orbital cortex, and right lateral occipital cortex differences when the seed was placed in the left DLPFC; and there were significant differences in the left subcallosal cortex, right lingual gyrus and left caudate when the seed was placed in the right DLPFC (P < 0.05, FDR corrected) (Table 2, Fig. 2A,B). Furthermore, there existed a severity-dependent decrease in the mean connectivity strength in the left precentral gyrus from healthy controls and NHE to CHE cirrhotics (Fig. 2C).

**Table 2:** Brain regions showing significant difference in functional connectivity.

	QFDR-corrected	Cluster size	Coordinate			F value	Harvard-Oxford Cortical/Subcortical
			X	Y	Z		Structural Atlas
dACC Left	0.989	11	−16	−2	−6	11.53	L. Pallidum
	0.989	14	−62	2	2	9.42	L. Precentral Gyrus
dACC Right	0.885	36	−20	58	32	11.05	L. Frontal Pole
	0.885	12	58	−34	−18	10.90	R. Inferior Temporal Gyrus
	0.990	13	−60	2	2	9.82	L. Precentral Gyrus
	0.990	12	−22	−70	30	9.32	L. Lateral Occipital Cortex
	0.990	13	−18	−60	60	8.60	L. Lateral Occipital Cortex
DLPFC Left	0.784	21	−18	16	−14	11.82	L. Frontal Orbital Cortex
	0.792	24	18	−76	44	10.33	R. Lateral Occipital Cortex
DLPFC Right	0.416	50	−8	14	−20	12.05	L. Subcallosal Cortex
	0.416	30	2	−68	−4	11.14	R. Lingual Gyrus
	0.416	21	−20	−10	26	10.94	L. Caudate

Seeds in dACC and DLPFC among normal controls, cirrhotics without hepatic encephalopathy, and cirrhotics with covert hepatic encephalopathy (p < 0.001, *cluster size* > 10); dACC: dorsal anterior cingulate cortex; DLPFC: Dorsal lateral prefrontal cortex; FDR: False Discovery Rate; L:left; R:right.

**Figure 3:**
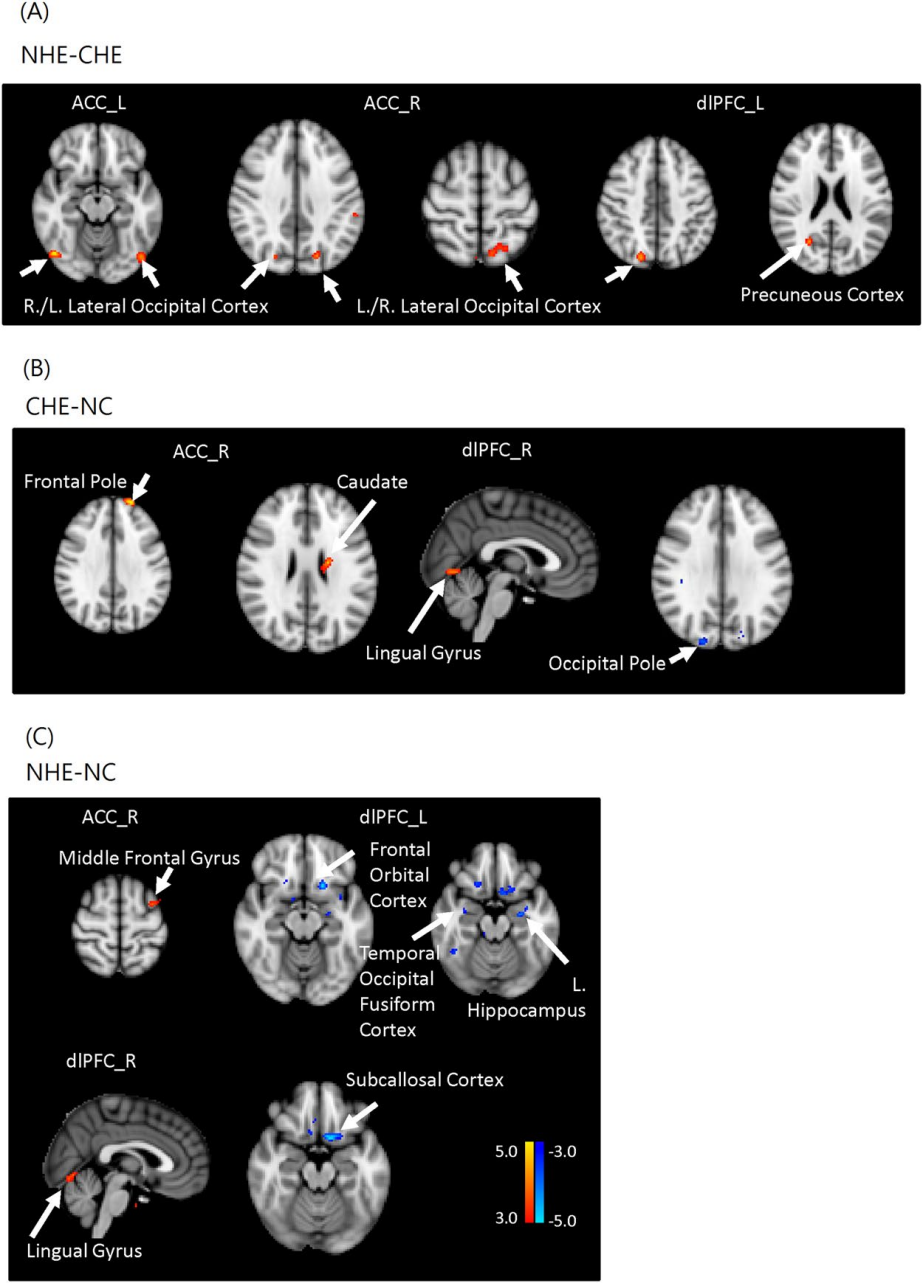
Post hoc between-group analysis of the differences in functional connectivity within the cognitive control network among groups. (**A**) NHE-CHE: Compared with CHE, NHE patients show higher connectivity between the bilateral dACC seed and lateral occipital cortex, and they show higher functional connectivity between the left DLPFC seed and the right lateral occipital cortex and the right precuneus cortex. (**B**) CHE-NC result: Compared with controls, CHE patients show lower connectivity between the right DLPFC and the right occipital pole, greater connectivity between the right dACC and the right frontal pole, and greater functional connectivity between the right DLPFC and the right caudate or right lingual gyrus. (**C**) NHE-NC: Compared with controls, NHE patients show greater connectivity between the right dACC seed and the left middle frontal gyrus, greater connectivity between the right DLPFC seed and the right lingual gyrus, lower connectivity between the left DLPFC seed and the left frontal orbital cortex, left hippocampus, right temporal occipital fusiform cortex, and the right frontal orbital cortex, as well as lower connectivity between the right DLPFC and the left subcallosal cortex. (all P < 0.05, false discovery rate corrected, dACC = dorsal anterior cingulate cortex; DLPFC = dorsolateral prefrontal cortex, NC = normal controls, NHE = non-hepatic encephalopathy cirrotics, CHE = covert hepatic encephalopathy).

For post-hoc analyses, the between-group FC differences within the CCN were compared. Relative to NHE cirrhotic patients, the CHE patients had lower FC in the bilateral lateral occipital cortex when the seed was placed in the bilateral dACC (P < 0.05, FDR corrected) and lower FC in the right lateral occipital cortex and the right precuneus cortex when the seed was placed in the left DLPFC (P < 0.05, FDR corrected) (Table 3, Fig. 3A). Relative to healthy controls, CHE patients had lower FC in the right occipital pole (P < 0.05, FDR corrected) when the seed was placed in the right DLPFC. CHE patients also had greater FC in the right frontal pole when the seed was placed in the right dACC and in the right caudate or right lingual gyrus when the seed was placed in the right DLPFC (P < 0.05, FDR corrected) (Table 3, Fig. 3B). Relative to healthy controls, NHE patients had greater FC in the left middle frontal gyrus when the seed was placed in the right dACC and in the right lingual gyrus when the seed was placed in the right DLPFC (P < 0.05, FDR corrected), while NHE patients had lower FC in the left frontal orbital cortex, left hippocampus, right temporal occipital fusiform cortex, and the right frontal orbital cortex when the seed was placed in the left DLPFC (all P < 0.05, FDR corrected) and in the left subcallosal cortex when the seed was placed in the right DLPFC (P < 0.05, FDR corrected) (Table 3, Fig. 3C).

**Table 3:** Brain regions showing significant difference within cognitive control network in any two groups.

	QFDR-corr	Cluster size	Coordinate			peak-T	Harvard-Oxford Cortical Structural Atlas
			X	Y	Z		
**NHE > CHE**							
dACC (L)	0.421	55	48	−70	−14	4.87	Lateral Occipital Cortex, inferior division
	0.421	63	−48	−76	−16	4.13	Lateral Occipital Cortex, inferior division
dACC (R)	0.417	50	46	−70	−14	4.65	Lateral Occipital Cortex, inferior division
	0.126	121	−20	−74	36	4.14	Lateral Occipital Cortex, superior division
	0.417	58	−10	−64	60	3.74	Lateral Occipital Cortex, superior division
DLPFC (L)	0.171	44	18	−76	44	4.21	Lateral Occipital Cortex, superior division
	0.171	32	24	−62	22	4.17	Precuneous Cortex
**NHE < CHE**							
	—	—	—	—	—	—	
**CHE > NC**							
dACC (R)	0.208	112	−20	58	32	4.97	Frontal Pole
DLPFC (R)	0.373	36	−20	−10	26	4.21	Caudate
	0.373	47	2	−68	−4	3.78	Lingual Gyrus
**CHE < NC**							
DLPFC (R)	0.493	50	18	−88	30	3.71	Occipital Pole
**NHE > NC**							
dACC (R)	0.536	20	−34	2	62	3.74	Middle Frontal Gyrus
DLPFC (R)	0.34	61	2	−70	−6	3.87	Lingual Gyrus
**NHE < NC**							
DLPFC (L)	0.595	67	−18	18	−14	4.71	Frontal Orbital Cortex
	0.595	46	−26	−10	−18	4.10	Left Hippocampus
	0.595	34	44	−50	−20	4.06	Temporal Occipital Fusiform Cortex
	0.595	31	22	22	−22	3.69	Frontal Orbital Cortex
DLPFC (R)	0.259	91	−10	12	−18	4.37	Subcallosal Cortex

Significant difference: p < 0.001, cluster size > 20; NC = normal control, NHE = non-hepatic encephalopathy cirrhotics, CHE = covert hepatic encephalopathy, dACC: dorsal anterior cingulate cortex; DLPFC: Dorsal lateral prefrontal cortex; FDR: False Discovery Rate; L:left; R:right.

### Correlations of FC in the CCN with PHES test

Pearson’s correlation analyses revealed that DST scores were positively correlated with the CCN FC in the left anterior supramarginal gyrus and the left inferior temporal gyrus; NCT-A scores were positively correlated with the CCN FC in the right inferior lateral occipital cortex, left superior lateral occipital cortex, left anterior supramarginal gyrus, right superior lateral occipital cortex, and right superior temporal gyrus; NCT-B scores were positively correlated with the CCN FC in the right inferior lateral occipital cortex, left inferior lateral occipital cortex, left superior lateral occipital cortex, right precuneus cortex, left inferior temporal gyrus, right superior lateral occipital cortex; LTT were scores positively correlated with the CCN FC in the left inferior lateral occipital cortex, left superior lateral occipital cortex, right precuneus cortex, left superior lateral occipital cortex, left inferior temporal gyrus, right superior lateral occipital cortex, and right superior temporal gyrus among cirrhotic patients. PHES total scores were positively correlated with the CCN FC in the bilateral lateral occipital cortex, right precuneus cortex, left inferior temporal gyrus, left supramarginal gyrus, and right superior temporal gyrus in the cirrhotic patient group (all P < 0.05, Table 4).

**Table 4:** Correlation between connectivities within cognitive control network and psychophysical performance.

Seed		Cognitive performance by PHES					
		DST	NCT-A	NCT-B	SDT	LTT	Total score
ACC right	R_Lateral Occipital Cortex, inferior division		R = 0.26, p = 0.01	R = 0.24, p = 0.03			R = 0.30, p = 0.005
	L_Lateral Occipital Cortex, inferior division,			R = 0.26, p = 0.01		R = 0.29, p = 0.007	R = 0.26, p = 0.01
ACC left	R_Lateral Occipital Cortex, inferior division						R = 0.25, p = 0.02
	L_Lateral Occipital Cortex, superior division		R = 0.31, p = 0.005	R = 0.23, p = 0.03		R = 0.41, p = 0.000	R = 0.32, p = 0.003
	R_Precuneous Cortex			R = 0.31, p = 0.004		R = 0.31, p = 0.005	R = 0.28, p = 0.01
	L_Lateral Occipital Cortex, superior division					R = 0.29, p = 0.007	R = 0.22, p = 0.04
	L_Inferior Temporal Gyrus, temporooccipital part	R = 0.23, p = 0.03		R = 0.25, p = 0.02		R = 0.27, p = 0.01	R = 0.30, p = 0.005
	L_Supramarginal gyrus, and anterior division	R = 0.32, p = 0.003	R = 0.28, p = 0.009				R = 0.26, p = 0.01
DLPFC right	R_Lateral Occipital Cortex, superior division		R = 0.23, p = 0.03	R = 0.28, p = 0.008		R = 0.28, p = 0.009	R = 0.33, p = 0.002
	R_precuneous Cortex					R = 0.38, p = 0.000	R = 0.26, p = 0.01
DLPFC left	R_Superior Temporal Gyrus,		R = 0.29, p = 0.007			R = 0.25, p = 0.02	R = 0.24, p = 0.02

Correlation between z value of functional connectivities within cognitive control network and psychophysical performance in PHES sub-domains and total scores in cirrhotic patients. *0.01 < p < 0.05; **0.01 < P < 0.005; ***0.001 < P < 0.005; dACC = dorsal anterior cingulate cortex, DLPFC: Dorsal lateral prefrontal cortex, PHES = Psychometric Hepatic Encephalopathy Score, DST = digit symbol test, NCT = number connection test, SDT = serial dotting test, LTT = line tracing test.

### Negative correlations of the CCN FC with venous ammonia and serum pro-inflammatory cytokine levels

Among the cirrhotic patients, the venous blood ammonia level was negatively correlated with the CCN FC in the right superior lateral occipital cortex; serum IL-6 level was negatively correlated with the CCN FC in the left superior lateral occipital cortex, left anterior supramarginal gyrus, right superior temporal gyrus, and right superior temporal gyrus; the serum TNF-alpha level was negatively correlated with the CCN FC in the right superior temporal gyrus (all P < 0.05, Table 5).

**Table 5:** Correlation between connectivities within cognitive control network and serum biochemistry levels.

Seed		Ammonia	IL-6	TNF-α
dACC left	L Lateral Occipital Cortex, superior division		R = −0.43, p = 0.007	—
	L Supramarginal gyrus, and anterior division		R = −0.32, p = 0.04	—
DLPFC right	R_Lateral Occipital Cortex, superior division	R = 0.27, P = 0.01		
DLPFC left	R Superior Temporal Gyrus, posterior division	R = −0.24 P = 0.03	R = −0.49, p = 0.002	R = −0.41, p = 0.01

Significant correlation between the z value of functional connectivities within cognitive control network and serum biochemistry levels (ammonia and proinlammatory cytokines) in cirrhotic patients. dACC = dorsal anterior cingulate cortex, DLPFC = Dorsal lateral prefrontal cortex, IL = interleukin, TNF = tumor necrosis factor.

## Discussion

To our knowledge, the present study was the first to identify impaired FC within the CCN among cirrhotic patients with CHE. Furthermore, disrupted FC was correlated with cognitive dysfunctions measured using PHES. In addition, blood ammonia and pro-inflammatory cytokines levels were negatively associated with the FC within the CCN among cirrhotics, suggesting the potential roles of the biochemical profiles in contributing to brain dysfunction.

Although the cirrhotic patients had decreased tendency of FC in the CCN, the cirrhotic patients and controls shared similar and consistent FC patterns in the CCN. These results demonstrated the reliability of these imaging data.

In the present study, we observed that the negativity of FC in the CCN was predominantly increased from NHE to CHE, particularly in the bilateral lateral occipital cortex, regardless of the seed placed in dACC or DLPFC. The lateral occipital cortex plays a key role in visual object processing^32^, and this region was significantly activated by attention^33^ and during object-based selection in previous functional MRI (fMRI) studies^34^. Previous studies have also shown that significantly increased local gyrification index in the lateral occipital cortex and the mean cortical thickness of the regions with structural abnormalities were negatively correlated with the PHES of patients with hepatitis B virus-related cirrhosis without overt HE^35^. In addition, judgement-related blood oxygenation level-dependent (BOLD) activation was decreased in CHE compared to controls in the right inferior parietal cortex (IPL). Furthermore, the analysis of psychophysiologic interaction suggests impaired neural interaction in patients with CHE, especially between the IPL and the parietooccipital cortex (Poc), the intraparietal sulcus, the ACC, the right PFC, the medial temporal lobe, and the extrastriate cortex V5^36^. In contrast, nonmanifest patients revealed an enhanced coupling between IPL and the postcentral cortex. These findings provide evidence of an early impaired and compensatory neural mechanism during visual judgement already in the mild stages of HE, which further suggest an aberrant coupling between cerebral regions in the dysmetabolic brain among CHE patients^36^. In the present study, we further observed that the FC of the CCN in the lateral occipital cortex was correlated with most subdomain and total scores of PHES, the golden standard test to detect CHE^37^. In addition, the FC of the CCN in the lateral occipital cortex was also correlated with the venous ammonia and serum IL-6 levels, the important markers for HE^38^. Moreover, we observed that the FC within the CCN in the lateral occipital cortex was preserved in NHE patients but reduced in CHE patients, suggesting an important role of the lateral occipital cortex in the differentiation of CHE from NHE and may contribute to CHE development.

We also observed that right precuneus connectivity within the CCN was significantly reduced from NHE to CHE when the seed was placed in the left DLPFC. Precuneus was primarily involved in visuospatial coordination, higher-order cognitive tasks, and conscious information processing^39^. It has reciprocal corticocortical connections with adjacent areas of the posteromedial cortex, namely the posterior cingulate and retrosplenial cortices, which involved spatial/memory functions. The precuneus is also selectively associated with other parietal areas involved in visuospatial information processing^40^. There were also extensive connections between the precuneus and the dorsal premotor area, the supplementary motor area (SMA) and the anterior cingulate cortex^41^. Similarly, the corticocortical projections from the precuneus to the lateral parietal areas and premotor cortex^42^ play a pivotal role in the motor coordination with visual guidance of hand movements^43^ and reaching^44^. Prior structural fMRI studies have identified significantly increased cortical thickness in the precuneus in HBV-related cirrhotics, suggesting low-grade brain edema in this region that potentially contributes to the impaired connectivity in the precuneus^35^. These findings are consistent with several studies showing decreased regional homogeneity in the precuneus and changes of amplitude of low frequency fluctuation and fraction in CHE patients^45,46^.

In the present study, we further observed that the lower FC in precuneus was associated with poor cognitive performance, indicated by PHES total scores and PHES-LTT subscores, referring to visuospatial tracing performance. Nagahama *et al*^47^. conducted an fMRI study showing that the precuneus may process not only spatial attention but also attention shift between object features. In addition, evidence from recent functional imaging studies indicated that the precuneus plays a crucial role in the internal mentation process of self-consciousness^48^. An interaction between the precuneus and prefrontal cortex has been postulated in states of consciousness characterized by a high level of reflective self-awareness^49^. Since the precuneus belongs may be part of a neural network subserving self-awareness, this finding may explain why CHE patients typically have poor insight into their neurological deficits^50^.

Severity-dependent changes in FC were observed in the left precentral gyrus confirming previous studies showing significantly decreased amplitude of low frequency fluctuation in the precentral gyrus among patients with CHE^45^. Therefore, the precentral gyrus may be important and discriminative brain regions responsible for CHE development^51^.

We observed that the serum levels of pro-inflammatory cytokines (IL-6 and TNF-alpha) and blood ammonia were negatively correlated with FC within the CCN. Specifically, FC within the CCN in the superior temporal gyrus (STG) was associated with IL-6, TNF-alpha, and ammonia. These findings echoed the results of recent studies showing STG thinning^52^ and reduced bilateral STG FC in CHE^21^. Such abnormalities might lead to the STG-mediated defect in auditory processing and social cognition processing among CHE patients. However, the IL-6 level was negatively correlated with FC within the CCN in the anterior supramarginal gyrus (aSMG), part of the somatosensory association cortex involved in the perception of space and extreme location, phonological and articulatory processing^53^. This finding is consistent with a recent fMRI study showing decreased connectivity between the right SMG and left thalamus in patients with CHE^54^. In summary, the negative correlation of FC with the level of IL-6, TNF-alpha and ammonia strengthens the role of systemic inflammation and hyperammonemia in the pathogenesis of cognitive impairment among cirrhotic patients with CHE^55^.

Although we identified a negative association between biochemical makers (ammonia and cytokines) and FC within the CCN, several studies have reported different results. For example, one study showed neither ammonia nor IL-6 levels in correlation with any signal changes in brain regions^56^. In contrast, the ammonia level was associated with FC within DAN^3^, DMN^3^, bilaterally auditory and somatosensory cortices^20^. However, studies have also reported that blood ammonia had no significant correlation with DMN^57^ and ACC^21^. The underlying causes of the wide discrepancy between the blood biochemistry and brain connectivity are still unknown. Although we have identified the correlation of different blood biomarkers to different loci of disruption of FC within the CCN, the interaction among different brain loci and the potential synergistic relationship between ammonia and pro-inflammatory cytokines remained unclear and warrant further research^3^.

The mean blood ammonia level among our enrolled cirrhotic patients were not high. ‘Cerebral’ ammonia play an important role in the pathogenesis of HE. However, ‘serum’ ammonia levels may not reliably correlate with HE symptoms or outcomes in chronic liver disease^58–60^. Therefore, in clinical practice, routine measurement of blood ammonia levels is not recommended as the results would not change the diagnosis nor management approach in HE patients^61,62^. Thus, the normoammonaemic status in our patients may reflect the fact that most of them were in the mild to moderate form of cirrhosis (Child A/B group). Actually, besides cerebral ammonia, recent studies also point to the important synergistic role of ‘inflammation’ in the HE pathogenesis^19^. Traditionally, low platelet count has been viewed as a marker in identifying portal hypertension in compensated liver cirrhosis^63^. Our cirrhotic patients have low platelet count and the CHE patients had even significant lower platelet count when compared with that in NHE counterpart, suggestive of the presence of liver failure/porto-systemic shunts. Since our CHE patients was older than the NHE ones, aging process and the associated co-morbidities in our cirrhotics might contribute to the observed neuropsychiatric abnormalities and disturbed neuro-network. However, no statistical difference in age, alcohol use, and level of depression and anxiety was noted among groups (Table 1, P > 0.05). Furthermore, as mentioned in the method, the patients with history of heart disease, respiratory system disease, renal failure and major neuropsychiatric diseases which would affect the target neuro-network were excluded. All these measurements might potentially minimize the bias from aging process and co-morbidities. Putting together, we believe that the observed neuropsychiatric abnormalities and disturbed neuro-network in our cirrhotic patients is derived from CHE *per se*. Nevertheless, unmeasured co-morbidities, such as pre-diabetic status, may still exist to affect the disturbed CCN in CHE patients.

The present study has several other limitations. First, although we observed deterioration in functional connectivity changes with the CCN during the development of CHE, we did not measure the changes of the CCN in overt HE patients. The patients with overt HE may display even more severe disconnection within the CCN, which deserves for future investigation even though these patients can be too uncooperative to cope with the resting fMRI study. Second, we selected bilateral dACC and DLPFC as the seeds rather than left or right subregions within the CCN in the present study, which might lead to a failure in identifying potentially important connectivity contributing to the CHE pathogenesis. Third, the present study did not evaluate the interference of structural changes within the CCN. Further studies incorporating structural imaging may provide more evidence to support these findings. We excluded the subjects with depressive symptoms to avoid the effect of mood on brain functional connectivity, which may limit the application of these results on cirrhotic patients with mood disturbances.

In conclusion, the present study demonstrated progressively disrupted FC in the CCN from cirrhotic patients without HE to the development of CHE, associated with cognitive dysfunction and increased ammonia/proinflammatory cytokine levels, which findings support an aberrant coupling between cerebral regions in the dysmetabolic brain among CHE patients. Impaired FC in the CCN may serve as a complementary biomarker for CHE.
